# Submarine Indoor Air Quality and Crew Health: A Critical Narrative State-of-the-Art Review of Respiratory and Cardiovascular Risks

**DOI:** 10.3390/toxics14010033

**Published:** 2025-12-27

**Authors:** Jérôme Sinquin, Aurélie Sachot, Fabrice Entine, Jean-Ulrich Mullot, Marco Valente, Samir Dekali

**Affiliations:** 1 Medical Service for the Nuclear-Powered Ballistic Missile Submarine Squadron (ESNLE), 29240 Brest, CEDEX 9, France; 2 Naval Techniques (DGA TN), Directorate General of Armaments, 83000 Toulon, France; 3 Central Direction of the Army Health Service (DCSSA), 75509 Paris, CEDEX 15, France; 4 Emerging Technologies Risk Unit, French Armed Forces Biomedical Research Institute (IRBA), BP73, 91223 Brétigny-sur-Orge, CEDEX, France

**Keywords:** submarine, indoor air quality, occupational exposure, respiratory health, cardiovascular health, VOCs, PM_2.5_, carbon dioxide, bioaerosols, oxidative stress

## Abstract

Background: Submarines represent extremely confined environments where breathing air is continuously recirculated for extended periods with minimal renewal, generating complex multipollutant atmospheres. Objectives: This critical narrative review aims to (i) summarize sources and composition of submarine indoor air, (ii) evaluate respiratory and cardiovascular risks for crews, and (iii) assess current purification technologies. Methods: A narrative review was conducted following PRISMA recommendations applicable to non-systematic reviews. The PubMed search covered all years from inception to September 2025, complemented by backward citation tracking and technical reports. Results: Eligible studies consistently report elevated levels of CO_2_, VOCs, NO_X_, CO, PM_2.5_, and bioaerosols aboard submarines. Evidence from submariner cohorts and toxicological studies indicates risks of airway irritation, impaired mucociliary defenses, endothelial dysfunction, cardiovascular stress, and neurobehavioral alterations. Conclusions: Submarine indoor air quality is a credible determinant of crew health. Existing filtration systems mitigate some risks but do not address multipollutant mixtures adequately. Improved real-time monitoring, advanced filtration, CFD-guided airflow optimization, and longitudinal medical surveillance are necessary.

## 1. Introduction

Submarines are extremely confined environments in which breathing air is largely recirculated for prolonged periods with little or no fresh-air exchange. Multiple onboard sources—including human metabolism, off-gassing from construction and furnishing materials, cooking and cleaning, maintenance and combustion processes, and microbial growth—continuously release contaminants, creating a complex multipollutant atmosphere that remains insufficiently characterized for modern platforms [1]. While historical efforts focused on vital and safety parameters (O_2_, CO_2_, H_2_, fire/smoke), the establishment of formal assessment programs has progressively recognized health implications that go beyond survivability to encompass chronic respiratory, cardiovascular, and neurobehavioral outcomes [1]. Early descriptive physiological work conducted during the first decades of prolonged submarine patrols had already raised hypotheses regarding potential metabolic, renal, and mineral homeostasis disturbances associated with long-term confinement and chronic hypercapnia [2].

Available measurements and extrapolations from analogous confined settings indicate that submarine indoor air typically includes carbon dioxide (CO_2_), nitrogen oxides (NO_x_), carbon monoxide (CO), volatile organic compounds (VOCs), fine and ultrafine particulate matter (PM), and bioaerosols. Documented contributors comprise propulsion/auxiliary systems, aging of polymeric materials, maintenance operations, personal care products, galley activities, and humid microclimates that favor microbial proliferation [3,4,5,6]. These emissions accumulate during prolonged submergence when air renewal is limited, and despite routine use of CO_2_ scrubbers, high-efficiency particulate air (HEPA) filtration, and activated carbon, current controls only partially address the breadth of gases, particles, and biological agents encountered under operational conditions [3,5].

From a health perspective, chronic low-to-moderate exposures are biologically plausible drivers of respiratory impairment—ranging from airway hyperreactivity and asthma to chronic obstructive patterns and fibrotic remodeling—and of cardiovascular dysfunction through endothelial impairment, oxidative stress, systemic inflammation, and autonomic imbalance. Observations in submariners and related occupational cohorts have reported elevated cardiovascular risk markers and sleep-disordered breathing under high ambient CO_2_, while toxicological studies in controlled atmospheres support multipollutant effects on pulmonary and neurobehavioral endpoints [7,8]. Importantly, cognitive performance decrements reported at low-to-moderate indoor CO_2_ in office-like settings may reflect both CO_2_ and co-varying VOC loads; experimental studies in submariners exposed acutely to 0.06–1.5% CO_2_ have not consistently demonstrated direct CO_2_ effects on decision-making, suggesting potential adaptation and/or confounding by ventilation-linked VOCs [9,10,11]. Together, these data argue for integrated consideration of multipollutant mixtures rather than single-agent thresholds.

Despite operational relevance, independent assessment is challenged by limited access to contemporary measurements, heterogeneity across classes and eras, and military confidentiality. Consequently, peer-reviewed evidence remains fragmented, with few longitudinal datasets linking time-resolved exposures to objective respiratory, cardiovascular, and neurocognitive outcomes in submariners [1]. This review, therefore, undertakes a critical state-of-the-art synthesis of submarine indoor air composition and crew health.

The objectives of this review are to:

(i) Summarize sources and composition of submarine indoor air;
(ii) Critically assess respiratory, cardiovascular, and neurobehavioral risks for crews;
(iii) Evaluate current air-treatment technologies and identify evidence gaps and priorities for mitigation and research.

We also identify evidence gaps and propose actionable directions—high-resolution real-time monitoring integrated with crew health metrics, next-generation multi-pollutant filtration/catalysis, airflow optimization via computational fluid dynamics, and tailored longitudinal medical surveillance—to inform evidence-based standards for safer undersea operations [1,3,5].

## 2. Methods

### 2.1. Review Approach

We performed a narrative search in PubMed only (from inception through September 2025), screened reference lists, and considered public technical reports. Priority was given to studies reporting measured onboard exposures, health outcomes in submariners, mechanistic insights from confined environments, and evaluations/limitations of air treatment systems [2,3,4,6,7,8,9,10]. Because of heterogeneity in designs, settings, and outcomes, we did not perform a meta-analysis or any quantitative pooling.

### 2.2. Synthesis and Certainty of Evidence

The literature search was performed exclusively in PubMed, from inception through September 2025. Boolean strings combined domain, exposure, and outcome terms (e.g., submarine AND (“indoor air” OR “air pollution” OR VOCs OR particulate matter OR carbon dioxide OR nitrogen oxides OR bioaerosols) AND (respiratory OR cardiovascular OR neurocognitive)). We complemented database results with backward citation tracking from included records and key technical reports. Run dates were logged internally (last update: September 2025); full queries are available from the corresponding author upon request.

### 2.3. Information Sources and Search Strategy

The literature search relied on PubMed as the primary database, which indexes the majority of operational and occupational studies relevant to submarines. Boolean search strings combined pollutant terms (CO_2_, VOCs, NO_x_, PM_2.5_), exposure descriptors (“indoor air”, “submarine”), and outcome terms (respiratory, cardiovascular, neurocognitive). The search included all years from database inception to September 2025. Although only PubMed was searched, this choice reflects the fact that most submarine-relevant medical and technical studies are indexed in this database. To broaden coverage, backward citation tracking was systematically applied to all included articles and key naval technical reports.

We included: (i) operational measurements aboard submarines or well-described analog confined environments informing exposure profiles, (ii) health studies in submariners, (iii) toxicological/mechanistic studies relevant to multipollutant mixtures, and (iv) evaluations or limitations of air-treatment systems. We excluded non-informative commentary, non-retrievable items, and animal-only studies without translational relevance.

### 2.4. Inclusion and Exclusion Criteria

**Figure 1:**
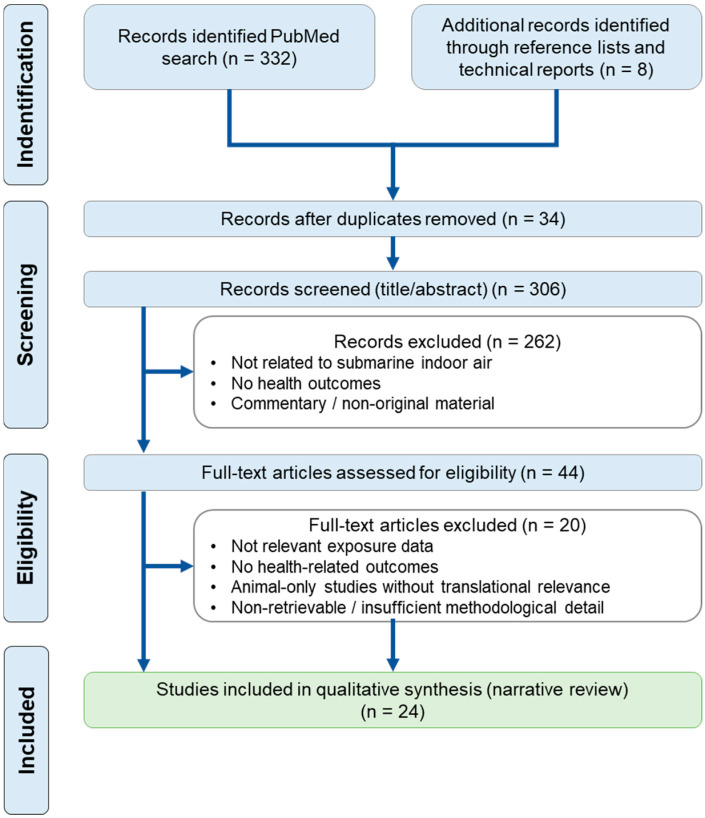
**PRISMA flow diagram of study selection.** Records were identified through a PubMed search (n = 332) and additional sources including reference lists and technical reports (n = 8). After duplicate removal, 306 records were screened based on titles and abstracts, leading to the exclusion of 262 records. Forty-four full-text articles were assessed for eligibility, of which 20 were excluded for predefined reasons. A total of 24 studies were included in the qualitative synthesis (narrative review).

The literature search, screening, and study selection process followed PRISMA principles adapted for narrative reviews and is summarized in the PRISMA flow diagram (Figure 1).

Records were screened at title/abstract and full-text stages against predefined criteria. For each included study, we charted design, platform/context, exposure metrics (gases, particles, bioaerosols), outcomes (respiratory, cardiovascular, neurobehavioral), and key limitations. Disagreements were resolved by discussion and consensus.

We included:

− Operational measurements aboard submarines or analogous confined environments;
− Submariner cohort or occupational studies;
− Mechanistic toxicology studies relevant to multipollutant mixtures;
− Evaluations of air-treatment systems.

We excluded:

− Commentary or non-original material;
− Non-retrievable items;
− Animal-only studies without translational relevance.

### 2.5. Study Selection and Data Charting

Because included studies differed considerably in design (operational measurements, epidemiology, toxicology), no single validated tool was applicable for quantitative quality scoring. Instead, we used a qualitative approach based on convergence across study types, internal validity, and applicability to contemporary submarine platforms.

### 2.6. Use of AI-Assisted Language Editing

A generative AI tool (ChatGPT, version 5.1, OpenAI, San Francisco, CA, USA) was used exclusively for English language polishing of draft sentences and paragraphs. No AI tool was used to identify, select, extract, analyze, or interpret data, nor to generate scientific content. All authors reviewed, edited, and approved the final text, and take full responsibility for the manuscript’s scientific integrity.

## 3. Results

### 3.1. Characteristics of Included Studies

A total of 24 studies were included after screening (Figure 1) in this narrative review, encompassing observational, cohort, cross-sectional, experimental, and registry-based designs conducted in military submarine populations or closely related confined-environment settings.

As summarized in Supplementary Table S1, most studies investigated active or former submariners from European and North American navies, with exposure generally defined as submarine service or confined submarine atmosphere. Only a limited subset of studies provided direct exposure measurements, mainly for carbon dioxide or incident-related diesel exhaust, while the majority relied on assumed or qualitative exposure characterization.

Health outcomes predominantly concerned respiratory symptoms and lung function, followed by cardiovascular mortality and risk markers, neurocognitive performance, and general health indicators. Comparator groups, when available, included surface naval personnel or the general population. Across studies, important limitations included exposure misclassification, healthy worker bias, cross-sectional designs, and limited longitudinal follow-up, which constrain causal inference.

### 3.2. Sources and Composition of Submarine Indoor Air

Submarine atmospheres rely on extensive air recirculation with minimal fresh-air exchange during submerged operations. Multiple onboard sources—human metabolism, materials off-gassing, galley and cleaning operations, maintenance/combustion processes, and microbial growth—create a multi-pollutant mixture whose composition and reactivity vary by platform, mission phase, and maintenance status [1,12]. Long patrols promote accumulation and chemical aging (secondary formation, sorption/desorption), while confidentiality and design heterogeneity across classes/eras hinder independent assessment and standard setting [1,13]. An overview of onboard contaminants, their sources, indicative magnitudes, available controls, and key health points is provided in Table 1.

Crew metabolism is the dominant continuous source of CO_2_. Chemical scrubbing constrains but does not eliminate chronically elevated levels relative to typical buildings; CO_2_ management underpins submariner guidance for Continuous Exposure Limits (CEL) and Emergency Exposure Limits (EEL) [1]. Proposed continuous limits around ~0.8% have been informed by developmental toxicology in rats and by risk-management frameworks that also consider acute exposure guideline levels [14,15]. The practical challenge is that CO_2_ often covaries with ventilation-sensitive co-pollutants (VOCs, moisture), so interpreting CO_2_ in isolation may be misleading for health-risk attribution [1].

Nuclear-powered submarines operate with continuous propulsion and minimal combustion-related emissions, resulting in more stable pollutant patterns dominated by CO_2_ accumulation and material off-gassing.

Classic diesel–electric submarines, especially during snorkeling or misstart events, exhibit transient but intense emissions of NO_x_, CO, PAHs, and ultrafine particles. Diesel exhaust exposures were also observed directly in submariners, consistent with real-world maintenance/harbor operations and transient events [15]. Diesel misstart events are recognized operational sources of PAHs and ultrafine particles. However, no peer-reviewed measurement datasets aboard submarines were identified in our search, highlighting a critical gap in exposure characterization. Targeted monitoring campaigns are needed to quantify PAH concentrations and related health implications.

Air-independent propulsion (AIP) refers to a recent propulsion technology that allows a non-nuclear submarine to operate without resorting to atmospheric oxygen (by surfacing or using a snorkel). AIP can supplement or replace the diesel–electric propulsion system of non-nuclear vessels and contributes to indoor pollution from NO_x_, CO, and fine particulate matter. Combustion and air-independent propulsion, plus maintenance activities and incident off-gassing, contribute NO_x_, CO, and fine particles; operational measurements on an AIP submarine documented these species as relevant exposures [3].

Ventilation dynamics and compartment pressures differ significantly between platforms and propulsion technology, influencing pollutant transport and crew exposure. Such gases may participate in secondary chemistry, adding to oxidant and particle formation under recirculation [12].

VOCs arise from polymeric/coating materials, adhesives/lubricants, personal-care products, galley/cleaning agents, and exhaled breath. Legacy shipboard pollutants (e.g., PCBs on older vessels) illustrate persistent material-derived hazards [4]. Breathomics/closed-habitat work highlights VOC complexity and turnover in recirculated atmospheres, with human emissions and activities as important modulators [5]. Naval/indoor epidemiology has long recognized activity/material-driven VOC burdens and respiratory impacts [16,17].

PM originates from mechanical wear/resuspension, galley/cleaning aerosols, and engine/auxiliary processes. HEPA captures particles but not co-occurring gases, so PM must be evaluated alongside NO_x_/VOCs. The cardiopulmonary relevance of PM_2.5_—including endothelial dysfunction, arrhythmia risk, and systemic inflammation—is well established [8,18,19], and exposure profiles aboard AIP platforms underscore the need for joint particle–gas controls [3]. Emerging maritime/indoor studies continue to quantify PM under variable ventilation and occupancy [20].

High occupancy and humid niches favor microbial persistence; classical submarine surveys reported airborne bacteria/fungi and crew carriage [6,21]. Recent naval experience with SARS-CoV-2 underscores transmission potential in tightly confined crews despite air treatment, emphasizing the role of ventilation patterns, filtration efficiency, and behavior in outbreak control [22].

CO_2_ scrubbing and related chemistries can introduce secondary exposures (e.g., amines/degradation products); a clinical report described new-onset asthma temporally linked to poor air quality with MEA use, motivating targeted monitoring/source characterization [23]. These risks interact with temperature/humidity and filter bed aging [12].

Mission duration (cumulative load), airflow compartmentalization/pressure regimes, maintenance cycles, and temperature/humidity control shape pollutant dynamics and mitigation performance. Current controls—CO_2_ scrubbing, HEPA, activated carbon—are selective, leaving gaps for gases (NO_x_, polar VOCs) and bioaerosols under real-world constraints [3,5,12]. Computational fluid dynamics (CFD) can reveal stagnation/recirculation zones that defeat nominal ventilation rates [24,25].

To enhance clarity and rigor, findings are presented by pollutant class and supported by operational measurements, epidemiological observations, and mechanistic evidence as available.

**Table 1:** **Submarine indoor air contaminants—sources, indicative concentrations, controls, and health relevance.** Principal chemical and biological contaminants reported in submarine environments. Indicative concentrations are order-of-magnitude ranges synthesized from submariner and other confined-environment literature; actual levels vary by platform, mission phase, and compartment. Controls listed are representative rather than exhaustive. Abbreviations: CO_2_ (carbon dioxide); CO (carbon monoxide); NO_x_ (nitrogen oxides); NO_2_ (nitrogen dioxide); O_3_ (ozone); VOCs (volatile organic compounds); BTEX (benzene, toluene, ethylbenzene, xylenes); PM_2.5_ (particulate matter < 2.5 μm); LiOH (lithium hydroxide); PTR-MS (proton-transfer-reaction mass spectrometry); UVGI (ultraviolet germicidal irradiation); HEPA (high-efficiency particulate air filter); CFU (colony-forming units); H_2_S (hydrogen sulfide); NH_3_ (ammonia); ppm (parts per million); ppb (parts per billion); μg m^−3^ (micrograms per cubic meter); CFU m^−3^ (colony-forming units per cubic meter).

Pollutant/Group	Main Onboard Sources	Indicative Magnitude	Existing Controls/Technologies	Key Health Points	Key References
CO_2_	Crew respiration; maintenance/combustion events	Hundreds to thousands of ppm in confined spaces	Scrubbers (amines, LiOH); ventilation; continuous monitoring	Decreased cognitive performance; headaches	[1,9,11,26,27,28]
CO	Combustion; maintenance; incidents	ppm	CO catalysts; detectors; operating procedures	Tissue hypoxia; headaches; nausea	[1,15,20]
NO_x_ (NO_2_)	Engines/snorkel; welding; cooking	ppb–ppm	Targeted ventilation; oxidation filters	Respiratory irritation; inflammation	[1,10]
O_3_	Electrical equipment; some air purifiers	ppb	Activated carbon; catalysis; source avoidance	Airway irritation	[1,10]
Aldehydes (e.g., formaldehyde)	Materials; glues; paints; cooking	ppb–low ppm	Activated carbon; selection of low-emitting materials	Irritation; sick-building-type symptoms	[1,10,14]
VOCs (BTEX, solvents)	Materials; maintenance; cleaning products	ppb–ppm	Activated carbon; source control; PTR-MS for tracking	Neurotoxicity; headaches	[1,5,10,25]
Particles (PM_2.5_/ultrafine)	Cooking; maintenance; abrasion	µg m^−3^ (variable)	Filtration (HEPA); source limitation	Cardiovascular and respiratory effects	[18,29,30]
NH_3_ H_2_S (occasional)	Sanitary areas; material degradation	ppb–ppm	Activated carbon; dedicated ventilation	Irritation; acute toxicity	[1,15]
Bioaerosols	Occupants; damp surfaces	CFU m^−3^ (variable)	Filtration/UVGI; hygiene; humidity control	Infections; allergens	[10,14,22]

### 3.3. Health Effects in Submariners

To orient the reader before the organ-specific subsections, Table 2 summarizes the main health effects and indicators reported across submariner and confined-environment literature.

#### 3.3.1. Respiratory Outcomes

Chronic low-to-moderate multi-pollutant exposures plausibly drive airway irritation/hyperreactivity, impaired mucociliary defenses, infection susceptibility, and progressive obstructive or fibrotic remodeling. Mixtures of VOCs-NO_x_/CO-PM_2.5_ activate oxidative and inflammatory pathways [8,18,19]. Operational diesel exposures have been described [31]; naval cohorts reported asthma and respiratory symptoms consistent with indoor/occupational determinants [16,17]. Submariners have shown sleep-related respiratory disturbances under elevated ambient CO_2_, aligning with hypercapnia-linked ventilatory control changes [7]. However, robust longitudinal datasets coupling time-resolved exposures and objective lung function or imaging remain scarce, reflecting measurement and access constraints [1,13].

#### 3.3.2. Cardiovascular Outcomes

Multiple mechanistic channels connect submarine pollutants to cardiovascular risk. Sustained hypercapnia can modulate endothelial nitric-oxide signaling and impair vascular reactivity, contributing to hypertensive phenotypes [28]. Complex combustion-derived mixtures—analogous to urban traffic emissions and composed of NO_x_, CO, VOCs, and fine particles —promote endothelial dysfunction, autonomic imbalance, and pro-arrhythmic states; volatile co-pollutants add oxidative burden [18,29]. Historical naval observations reported higher prevalence of hypertension and risk markers in submariners vs. other personnel, arguing for tailored cardiovascular surveillance [7,32]. Large retrospective cohort analyses in U.S. Navy submariners did not report excess all-cause or cardiovascular mortality compared with the general population, highlighting a pronounced healthy worker effect despite prolonged occupational confinement [33]. Beyond single-organ cardiovascular endpoints, a large cross-sectional study conducted in Korean naval personnel reported a significantly higher prevalence of multimorbidity among submariners compared with non-submariners, even after adjustment for age, smoking, alcohol consumption, and naval rank, suggesting a broader systemic health burden associated with submarine service [34]. Operational stressors—sleep/circadian disruption, inactivity, psychosocial strain—likely synergize with environmental burdens during patrols [35,36].

#### 3.3.3. Neurobehavioral Outcomes

Maintaining cognition is safety-critical. Office-like experiments reported decision-making decrements at low-to-moderate indoor CO_2_ [9,26], and a short review summarized building-related symptoms in the 500–1000 ppm range [10]. In contrast, submariner-specific CO_2_ exposures (0.06–1.5%, ≈600–15,000 ppm) reported no measurable decision-making impairment, suggesting adaptation and/or confounding by ventilation-linked co-pollutants [11,37]. Low-ventilation decrements often track VOCs more closely than CO_2_ alone [26]. Fatigue, isolation, and noise are additional, non-chemical degraders of vigilance/executive function during patrols [32,36].

#### 3.3.4. Other Effects and Knowledge Gaps

Recent survey-based data suggest that, despite environmental and operational constraints, self-reported vitality and general health indicators may be preserved or even higher in experienced submariners compared with the general population, potentially reflecting selection and adaptation effects [38]. Concerns extend to acid-base balance and renal bicarbonate handling, endocrine/autonomic modulation, infection dynamics in crowded berthing, and potential gene–environment interactions in susceptible subgroups [39,40,41]. Proposed submariner CO_2_ limits derive partly from animal developmental toxicity and may not reflect multi-pollutant or endocrine endpoints [14]. Critical gaps include the paucity of time-aligned exposure–response datasets, limited access to contemporary operational measurements, and heterogeneity across platforms/eras [1,12].

**Table 2:** **Health effects of submarine indoor air exposures—indicators and strength of evidence.** Decision-oriented summary of reported health outcomes in submariners and related confined-environment literature. Rows group effects by organ system; columns list sentinel clinical outcomes/biomarkers and an evidence rating. Evidence levels: High—multiple controlled studies and/or convergent epidemiological/mechanistic data; Moderate—signals present but dependent on mixture, exposure level, or study limitations. Abbreviations: CO_2_ (carbon dioxide); CO (carbon monoxide); NO_2_ (nitrogen dioxide); O_3_ (ozone); VOCs (volatile organic compounds); PM_2.5_ (particulate matter < 2.5 μm); FeNO (fractional exhaled nitric oxide); CRP (C-reactive protein); FEV_1_ (forced expiratory volume in 1 s); HRV (heart-rate variability); CNS (central nervous system); AEGL (Acute Exposure Guideline Level); CEGL (Continuous Exposure Guidance Level); IAQ (indoor air quality); H_2_S (hydrogen sulfide); NH_3_ (ammonia). Symbol: ↓ denotes decrease.

Effect/Organ System	Indicators (Clinical/Biomarkers)	Strength of Evidence	Key References
CO_2_-related cognitive impairments	Decision/strategy scores; vigilance; simulated task performance	High (controlled trials + data from confined environments)	[9,11,20,26,28]
Respiratory irritation and inflammation (NO_2_, O_3_, aldehydes)	Symptoms (cough, irritation); FeNO; CRP; ↓ FEV_1_	High (reviews and IAQ guidelines)	[1,10,14]
Cardiovascular effects (PM_2.5_)	Blood pressure; heart-rate variability; inflammatory/oxidative markers	High (mechanistic and epidemiology reviews)	[18,29,30]
Solvent neurotoxicity (VOCs)	Neurobehavioral tests; CNS symptoms	Moderate (depends on mixture and exposure)	[5,25]
Non-specific symptoms (headache, fatigue)	Self-reported questionnaires; symptom scales	Moderate	[10,22,42]
Acute risks (CO, H_2_S, NH_3_)	Acute poisoning; neurological signs	High (AEGL/CEGL)	[1,15]

### 3.4. Current Air-Quality Controls and Their Limitations

Chemical absorbents effectively reduce metabolic CO_2_ but do not remove co-occurring gases or particles; regenerable sorbents (e.g., polyamines) aim to improve sustainability/footprint yet face throughput, humidity, and reliability constraints in operational settings [27,43,44]. Potential secondary exposures (amines/by-products) warrant surveillance in modern systems [23].

HEPA efficiently captures PM and some bioaerosols but is ineffective for most gases (VOCs, NO_x_, CO). Under recirculation, gas-phase accumulation persists despite good particulate removal, especially when humidity is high [1,3]. Activated-carbon stages offer complementary gas-phase control but exhibit finite capacity and humidity-dependent performance, necessitating predictive maintenance and breakthrough monitoring [12].

Multi-functional beds pairing HEPA + activated carbon + catalytic media improve coverage across particles + gases; sensor-assisted maintenance can avoid silent breakthrough [27,43,45]. Oxidation/catalysis modules, membrane-based NO_x_ removal, and humidity-robust sorbents are active development areas [44].

Electrolysis sustains oxygen but does not remove contaminants; incident risks constrain some chemical O_2_ systems. Control logic, therefore, decouples oxygenation from depollution and requires independent monitoring of air quality [1].

Despite filtration, persistent microbial flora can be detected in humid, poorly ventilated niches; targeted hygiene, moisture control, and air handling remain necessary [6,21].

Sparse real-time metrology and confidentiality impede performance tracking and independent appraisal. Existing systems, thus, provide partial protection against chemical/biological complexity, reinforcing the need for integrated strategies and clearer data-governance practices [1,5,46].

## 4. Discussion

This review highlights convergent evidence that multipollutant exposures aboard submarines are biologically plausible contributors to respiratory and cardiovascular alterations through mechanisms involving oxidative stress, low-grade inflammation, autonomic imbalance, and endothelial dysfunction [2,33,47]. Importantly, the available human data do not point to a single dominant toxicant, but rather to a chronic, multifactorial exposure profile combining hypercapnia, combustion-related gases, volatile organic compounds, fine and ultrafine particles, and operational stressors. Individually, these factors often remain below conventional occupational limits; collectively, they define a persistent exposure scenario with limited recovery time, which is not adequately captured by standard risk assessment paradigms [5,11].

Across epidemiological and observational studies in submariners, overt excess mortality or severe clinical disease is not consistently observed, and several cohorts illustrate a pronounced healthy worker effect [33,47]. However, this apparent absence of major health outcomes should not be interpreted as evidence of safety. Subtle but coherent signals—such as increased prevalence of respiratory symptoms, elevated cardiovascular risk markers, sleep-disordered breathing, or multimorbidity—suggest that current endpoints may lack sensitivity to detect long-term or cumulative effects [7,16,34,38]. These observations are consistent with findings from other confined or occupational environments, where chronic exposure to complex pollutant mixtures has been associated with functional alterations in the absence of overt disease [9].

A key challenge highlighted by this review lies in the mismatch between submarine exposure profiles and existing occupational exposure frameworks. Traditional threshold limit values (TLV) and maximum acceptable concentrations (MAC) are derived for 8 h work shifts with defined recovery periods, assumptions that do not apply to submerged operations. Continuous Exposure Limits (CEL) and Emergency Exposure Limits (EEL) provide more relevant guidance for submarine contexts, yet they remain largely substance-specific and do not fully account for multipollutant interactions or co-exposures characteristic of submarine atmospheres [2,48]. Evidence from other occupational settings further indicates that reliance on single-substance limits may underestimate risk in environments dominated by combustion-derived mixtures, including polycyclic aromatic hydrocarbons [49].

The limited strength of causal inference in the submarine health literature primarily reflects structural and operational constraints rather than a lack of biological plausibility. Access to contemporary exposure measurements is restricted by military confidentiality, exposure characterization is frequently indirect or assumed, and longitudinal designs linking time-resolved environmental data to objective health outcomes remain scarce [17,31]. In addition, heterogeneity across submarine classes, propulsion systems, mission profiles, and air-treatment technologies further complicates cross-study comparisons and limits external validity [5].

Taken together, the available evidence supports a precautionary interpretation: submarine indoor air should be considered a chronic multipollutant exposure environment, where health risks may arise from cumulative and interactive effects rather than from exceedance of individual thresholds. This perspective underscores the need to move beyond compliance-based monitoring toward integrated approaches combining high-resolution environmental measurements, biomarkers of exposure and early effect, and longitudinal medical surveillance [11,47]. Such advances are essential to refine risk assessments, inform adaptive exposure guidelines, and ultimately enhance crew protection under prolonged submerged conditions.

This review highlights convergent evidence that multipollutant exposures aboard submarines affect respiratory and cardiovascular physiology via oxidative stress, inflammation, and endothelial dysfunction. These findings align with observations in other confined environments but remain constrained by limited operational measurements and platform heterogeneity.

## 5. Future Directions and Mitigation Strategies

**High-resolution, real-time monitoring linked to health.** Deploy distributed, ruggedized sensor networks for CO_2_/NO_x_/CO/VOCs/PM with automated QA/QC, drift control and data governance and link exposures to operational or wearable health metrics to enable proactive interventions. Closed-habitat and breathomics insights support multi-analyte approaches [5,20,39,50]. Emerging naval case learning from respiratory virus management also argues for integrated environmental–medical dashboards [22].

Next-generation multi-pollutant purification. Combine HEPA with activated carbon and catalytic media (humidity-robust, regenerable) to address gases + particles simultaneously and adopt predictive maintenance to avoid breakthrough [27,43,45]. Materials/controls should be vetted for secondary emissions (amines/by-products) and monitored in situ [23].

**Airflow optimization and compartment strategy.** Use computational fluid dynamics (CFD) to map stagnation/recirculation, redesign diffusers/returns and compartment linkages (while respecting casualty-control constraints), and deploy mobile ventilation where needed. CFD-guided design complements filtration by reducing local accumulation and exposure heterogeneity [12,24,25].

**Longitudinal medical surveillance tailored to submariners.** Institute periodic assessment of respiratory, cardiovascular, and neurocognitive endpoints linked to exposure logs; integrate omics profiling to detect subclinical alterations with cumulative exposures; embed preventive cardiology/sleep-health programs for high-risk crew [7,30,32,36,40,41]. Occupational readiness frameworks argue for combining surveillance with targeted risk-reduction interventions [47,51].

**Governance and transparency.** Broaden access to de-identified exposure datasets for independent evaluation and standard setting; codify action levels and response playbooks that integrate sensor alerts, operational mitigations, and medical triage to sustain health and readiness [1,12,42,52,53]. International defense groups such as the Submarine Escape and Rescue Working Group (SMERWG) or other NATO bodies could coordinate the development of harmonized air-quality standards and operational guidance. Such coordinated, multinational frameworks would facilitate data sharing, methodological harmonization, and the translation of emerging evidence into operationally relevant standards across submarine fleets.

## 6. Conclusions

Submarine indoor air constitutes a recirculated, multipollutant environment in which metabolic carbon dioxide, combustion-related gases and particles, volatile organic compounds, and bioaerosols co-accumulate during prolonged submerged operations. Available operational measurements, historical surveys, and committee guidance consistently describe this exposure profile and indicate that current air-treatment systems—CO_2_ scrubbing, particulate filtration, and activated carbon—mitigate selected contaminants but do not comprehensively address combined chemical and biological exposures under real operational conditions [2,5,6,17].

Although the epidemiological evidence does not demonstrate clear excess mortality among submariners, convergent data support biological plausibility for adverse respiratory and cardiovascular effects associated with chronic exposure to complex pollutant mixtures, including oxidative stress, airway irritation, endothelial dysfunction, and autonomic imbalance [7,16,33,34,47]. Neurocognitive effects remain nuanced, with discrepancies between office-like exposure studies and submariner-specific experiments underscoring the importance of considering adaptation and co-pollutant interactions rather than carbon dioxide in isolation [9,11].

Overall, submarine indoor air quality should be regarded as a credible and potentially preventable determinant of crew health and readiness. Transitioning from single-constituent management toward integrated multipollutant monitoring, exposure-informed health surveillance, and improved air-treatment strategies represents a necessary step to strengthen evidence-based standards and enhance long-term protection of submarine crews [38,48] see Table 3.

## Figures and Tables

**Table 3:** **Operational controls for submarine indoor air-actions, targets, TRL, benefits, and constraints.** Decision-oriented summary mapping engineering and administrative actions to their primary exposure targets (gases, particles, bioaerosols), indicative technology readiness level (TRL), expected benefits, and key implementation caveats. TRL follows a 1–9 scale (9 = fully deployed). Abbreviations: CO_2_ (carbon dioxide); CO (carbon monoxide); NO_2_ (nitrogen dioxide); O_3_ (ozone); VOCs (volatile organic compounds); PM_2.5_ (particulate matter < 2.5 μm); HEPA (high-efficiency particulate air [filter]); UVGI (ultraviolet germicidal irradiation); IAQ (indoor air quality); LiOH (lithium hydroxide); HSE (health, safety, and environment). Symbol: ↓ denotes decrease.

Proposed Action	Target (Gas/PM/Bio)	Maturity/TRL	Expected Benefit	Constraints/Caveats	Key References
CO_2_ scrubbing (amines, LiOH) and setpoint optimization	CO_2_	High (TRL 8–9)	Lower ambient CO_2_; improved cognition	Regeneration/consumables; HSE management	[1,11,27]
Particulate filtration (HEPA) + source control	PM	High	↓ PM_2.5_; ↓ inflammatory markers	Filter maintenance; pressure drop	[18,29]
VOCs adsorption (activated carbon) + selection of low-emitting materials	VOCs/aldehydes	High/Moderate	↓ VOC peaks; ↓ symptoms	Saturation; media selection; monitoring	[5,10,14]
Continuous IAQ monitoring (CO_2_, CO, NO_2_/O_3_, VOCs, PM) with action thresholds	All	High	Early detection; control of ventilation/scrubbing	Calibration; data management	[1,5,28]
Operational protocols (galley ventilation, maintenance, hygiene)	PM/VOCs/bioaerosols	High	Reduced emissions and micro-sources	Adherence; training	[10,22,42]
Air hygiene (targeted UVGI; humidity control)	Bioaerosols	Moderate	Lower microbial load	Integration; UV safety	[14,22]

## Data Availability

Data sharing is not applicable to this article as no datasets were generated or analyzed during the current study. All information discussed is derived from published sources cited in the manuscript.

## Supplementary Materials

The following supporting information can be downloaded at: https://www.mdpi.com/article/10.3390/toxics14010033/s1, Table S1. PECO(TS) characteristics of studies included in the narrative review on submarine indoor air quality and crew health.

## Abbreviations

The following abbreviations are used in this manuscript:

AEGL	Acute Exposure Guideline Level
AIP	Air-independent propulsion
CEL	Continuous Exposure Limit (submariner guidance)
CEGL	Continuous Exposure Guidance Level
CFD	Computational fluid dynamics
CFU	Colony-forming units
CNS	Central nervous system
CO	Carbon monoxide
CO_2_	Carbon dioxide
COVID-19	Coronavirus disease 2019
CRP	C-reactive protein
EEL	Emergency Exposure Limit
FeNO	Fractional exhaled nitric oxide
FEV_1_	Forced expiratory volume in 1 s
HEPA	High-Efficiency Particulate Air (filter)
HSE	Health, safety, and environment
HRV	Heartrate variability
IAQ	Indoor air quality
LiOH	Lithium hydroxide
MEA	Monoethanolamine
NO	Nitric oxide
NO_2_	Nitrogen dioxide
NO_x_	Nitrogen oxides
O_2_	Oxygen
O_3_	Ozone
PCB	Polychlorinated biphenyls
PM	Particulate matter
PM_2.5_	Particulate matter < 2.5 µm
PPB/PPM	Parts per billion/parts per million (*v*/*v* unless stated)
PRISMA	Preferred Reporting Items for Systematic Reviews and Meta-Analyses
PTR-MS	Proton-transfer-reaction mass spectrometry
RH	Relative humidity
SARS-CoV-2	Severe Acute Respiratory Syndrome Coronavirus 2
SOP	Standing operating procedures (for AEGL development)
TRL	Technology readiness level
TVOC	Total volatile organic compounds
UFP	Ultrafine particles (<100 nm)
VOC/VOCs	Volatile organic compounds

## References

[1] National Research Council (US) Committee on Emergency and Continuous Exposure Guidance Levels for Selected Submarine Contaminants (2009). Emergency and Continuous Exposure Guidance Levels for Selected Submarine Contaminants: Volume 3.

[2] Davies D.M. (1973). Sixty Days in a Submarine: The Pathophysiological and Metabolic Cost. J. R. Coll. Physicians Lond..

[3] Persson O., Ostberg C., Pagels J., Sebastian A. (2006). Air Contaminants in a Submarine Equipped with Air Independent Propulsion. J. Environ. Monit. JEM.

[4] Still K.R., Arfsten D.P., Jederberg W.W., Kane L.V., Larcom B.J. (2003). Estimation of the Health Risks Associated with Polychlorinated Biphenyl (PCB) Concentrations Found Onboard Older U.S. Navy Vessels. Appl. Occup. Environ. Hyg..

[5] Pleil J.D., Hansel A. (2012). Submarines, Spacecraft and Exhaled Breath. J. Breath Res..

[6] Morris J.E. (1972). Microbiology of the Submarine Environment. Proc. R. Soc. Med..

[7] Margel D., White D.P., Pillar G. (2003). Long-Term Intermittent Exposure to High Ambient CO_2_ Causes Respiratory Disturbances during Sleep in Submariners. Chest.

[8] Hardt D.J., James R.A., Gut C.P., McInturf S.M., Sweeney L.M., Erickson R.P., Gargas M.L. (2015). Evaluation of Submarine Atmospheres: Effects of Carbon Monoxide, Carbon Dioxide and Oxygen on General Toxicology, Neurobehavioral Performance, Reproduction and Development in Rats. I. Subacute Exposures. Inhal. Toxicol..

[9] Satish U., Mendell M.J., Shekhar K., Hotchi T., Sullivan D., Streufert S., Fisk W.J. (2012). Is CO_2_ an Indoor Pollutant? Direct Effects of Low-to-Moderate CO_2_ Concentrations on Human Decision-Making Performance. Environ. Health Perspect..

[10] Azuma K., Kagi N., Yanagi U., Osawa H. (2018). Effects of Low-Level Inhalation Exposure to Carbon Dioxide in Indoor Environments: A Short Review on Human Health and Psychomotor Performance. Environ. Int..

[11] Rodeheffer C.D., Chabal S., Clarke J.M., Fothergill D.M. (2018). Acute Exposure to Low-to-Moderate Carbon Dioxide Levels and Submariner Decision Making. Aerosp. Med. Hum. Perform..

[12] Mazurek W. (1998). Current Submarine Atmosphere Control Technology. Life Support Biosph. Sci. Int. J. Earth Space.

[13] National Research Council (US) Committee on Emergency and Continuous Exposure Guidance Levels for Selected Submarine Contaminants (2009). Introduction. Emergency and Continuous Exposure Guidance Levels for Selected Submarine Contaminants: Volume 3.

[14] Howard W.R., Wong B., Yeager K.S.B., Stump D.G., Edwards T., Arden James R., Goodwin M.R., Gargas M.L. (2019). Submarine Exposure Guideline Recommendations for Carbon Dioxide Based on the Prenatal Developmental Effects of Exposure in Rats. Birth Defects Res..

[15] National Research Council (US) Subcommittee on Acute Exposure Guideline Levels (2001). Standing Operating Procedures for Developing Acute Exposure Guideline Levels for Hazardous Chemicals.

[16] Sims J.R., Tibbles P.M., Jackman R.P. (1999). A Descriptive Analysis of Asthma in the U.S. Navy Submarine Force. Aviat. Space Environ. Med..

[17] Ferrari M., Lodola L., Ghittori S., Zadra P., Ricciardi L., Imbriani M. (2005). Indoor air quality in an Italian military submarine. G. Ital. Med. Lav. Ergon..

[18] Hamanaka R.B., Mutlu G.M. (2018). Particulate Matter Air Pollution: Effects on the Cardiovascular System. Front. Endocrinol..

[19] Verma V., Sharma A., Anand N. (2021). Effect of Submarine Microclimate on Respiratory Physiology of Submariners: An Observational Study. J. Mar. Med. Soc..

[20] Stephens B., Kang I., Jagota K., Elfessi Z., Karpen N., Farhoodi S., Heidarinejad M., Rubinstein I. (2025). Study Protocol for a 1-Year, Randomized, Single-Blind, Parallel Group Trial of Stand-Alone Indoor Air Filtration in the Homes of US Military Veterans with Moderate to Severe COPD in Metropolitan Chicago. Trials.

[21] Morris J.E., Fallon R.J. (1973). Studies on the Microbial Flora in the Air of Submarines and the Nasopharyngeal Flora of the Crew. J. Hyg..

[22] Weinberg D., Edgar M., Majewski M., Wakefield R. (2022). Coronavirus Disease 2019 On Board a Submarine: A Retrospective Review. Mil. Med..

[23] Kraus R., Rose C.S., Meehan R.T. (2022). New Onset Asthma in a Nuclear Submariner Following Prolonged Exposure to Poor Air Quality.

[24] Fathi A.R., Yang C., Bakhtian K.D., Qi M., Lonser R.R., Pluta R.M. (2011). Carbon Dioxide Influence on Nitric Oxide Production in Endothelial Cells and Astrocytes: Cellular Mechanisms. Brain Res..

[25] Maculewicz E., Pabin A., Dziuda Ł., Białek M., Białek A. (2023). Selected Exogenous (Occupational and Environmental) Risk Factors for Cardiovascular Diseases in Military and Aviation. J. Clin. Med..

[26] Satish U., Cleckner L., Vasselli J. (2013). Impact of VOCs on Decision Making and Productivity. Intell. Build. Int..

[27] Goeppert A., Zhang H., Czaun M., May R.B., Prakash G.K.S., Olah G.A., Narayanan S.R. (2014). Easily Regenerable Solid Adsorbents Based on Polyamines for Carbon Dioxide Capture from the Air. ChemSusChem.

[28] Walsh H.J., Junejo R.T., Lip G.Y.H., Fisher J.P. (2024). The Effect of Hypertension on Cerebrovascular Carbon Dioxide Reactivity in Atrial Fibrillation Patients. Hypertens. Res..

[29] Bhatnagar A. (2022). Cardiovascular Effects of Particulate Air Pollution. Annu. Rev. Med..

[30] Jimenez M.T.B., Hahad O., Kuntic M., Daiber A., Münzel T. (2022). Noise, Air, and Heavy Metal Pollution as Risk Factors for Endothelial Dysfunction. Eur. Cardiol..

[31] Duplessis C.A., Gumpert B. (2008). Inhalational Diesel Exhaust Exposure in Submariners: Observational Study. Mil. Med..

[32] Vaernes R.J., Sandal G. (2003). Human Reactions to Deep-Water Conditions. Lancet.

[33] Burr R.G., Palinkas L.A. (1987). Health Risks among Submarine Personnel in the U.S. Navy, 1974–1979. Undersea Biomed. Res..

[34] Kang J., Song Y.-M. (2017). The Association between Submarine Service and Multimorbidity: A Cross-Sectional Study of Korean Naval Personnel. BMJ Open.

[35] Holy X., Bégot L., Renault S., Butigieg X., André C., Bonneau D., Savourey G., Collombet J.-M. (2015). Seasonal Influence over Serum and Urine Metabolic Markers in Submariners during Prolonged Patrols. Physiol. Rep..

[36] McGraw L.K., Turner B.S., Stotts N.A., Dracup K.A. (2008). A Review of Cardiovascular Risk Factors in US Military Personnel. J. Cardiovasc. Nurs..

[37] Scully R.R., Basner M., Nasrini J., Lam C.-W., Hermosillo E., Gur R.C., Moore T., Alexander D.J., Satish U., Ryder V.E. (2019). Effects of Acute Exposures to Carbon Dioxide on Decision Making and Cognition in Astronaut-like Subjects. NPJ Microgravity.

[38] Houtkooper A., Wingelaar T.T., Endert E.L., van Ooij P.-J.A. (2023). Self-Reported Vitality and Health Status Are Higher in Dutch Submariners than in the General Population. Diving Hyperb. Med..

[39] Cui F., Sun Y., Xie J., Li D., Wu M., Song L., Hu Y., Tian Y. (2023). Air Pollutants, Genetic Susceptibility and Risk of Incident Idiopathic Pulmonary Fibrosis. Eur. Respir. J..

[40] Nasser A., Bhutani S. (2017). Occupational Health Concerns: An Analysis of Physical Activity of Submariners. Med. J. Armed Forces India.

[41] Korhan O. (2019). Indoor Environment and Health.

[42] Sinquin J., Olesinski B., Agousty M., Couchouron G., Désidéri-Vaillant C., Breil D., Colléony T., Entine F., Mullot J.-U., Oulié C. (2025). Évaluation de l’exposition Au Chrome Hexavalent à Bord d’un Sous-Marin Nucléaire. Arch. Mal. Prof. Environ..

[43] Carey R., Gomezplata A., Sarich A. (1983). An Overview into Submarine CO_2_ Scrubber Development. Ocean Eng..

[44] Rau G.H. (2011). CO_2_ Mitigation via Capture and Chemical Conversion in Seawater. Environ. Sci. Technol..

[45] Mahmood R., Patel M.A., Anamika F.N.U., Shaik T., Aggarwal P., Garg N., Kamboj S.S., Gupta V., Jain R. (2024). Navigating the Depths of Cardiovascular Effects on Submariners. Cardiol. Rev..

[46] Goodall A. (2012). The Development of a Non-Powered Oxygen Generator for Royal Navy Submarines. Proceedings of the 42nd International Conference on Environmental Systems.

[47] Strand L.A., Rudvin I., Martinsen J.I., Fadum E.A., Grimsrud T.K. (2025). Cancer Incidence and Cause-Specific Mortality in 2663 Male Submariners with Service in the Royal Norwegian Navy between 1942 and 2005: A Registry-Based Cohort Study. BMJ Open.

[48] Drolet D., Thierry P.-R. (2007). Ajustement d’une VEMP Du RSST En Fonction d’un Horaire Non Conventionnel. https://pharesst.irsst.qc.ca/utilitaires/2/.

[49] Moen B.E., Nilsson R., Nordlinder R., Ovrebø S., Bleie K., Skorve A.H., Hollund B.E. (1996). Assessment of Exposure to Polycyclic Aromatic Hydrocarbons in Engine Rooms by Measurement of Urinary 1-Hydroxypyrene. Occup. Environ. Med..

[50] Beardslee L.A., Casper E.T., Lawson B.D. (2021). Submarine Medicine: An Overview of the Unique Challenges, Medical Concerns, and Gaps. Undersea Hyperb. Med..

[51] Abbasi A., Mirekhtiary F., Khandaker M.U., Maden S., Ali A.H. (2025). Radiological and Ecological Consequences of a Hypothetical Nuclear Submarine Accident in the Eastern Mediterranean. J. Radioanal. Nucl. Chem..

[52] Tappan D.V., Mooney L.W., Jacey M.J., Heyder E. (1979). Cardiovascular Risk Factors in Submariners. Undersea Biomed. Res..

[53] Tappan D.V., Weybrew B.B. (1982). Relationship of Personality Factors and Some Social Habits to Cardiovascular Risk in Submariners. Aviat. Space Environ. Med..

