# Supplementary material for: Submarine Indoor Air Quality and Crew Health: A Critical Narrative State-of-the-Art Review of Respiratory and Cardiovascular Risks
Source: Toxics. 2025 Dec 27;14(1):33. doi: 10.3390/toxics14010033 (PMC12845886; doi:10.3390/toxics14010033)
Supplement: Supplementary file 1 [file toxics-14-00033-s001.zip › toxics-4002122-supplementary.pdf]

Supplementary Table S1. PECO(TS) characteristics of studies included in the narrative review on submarine indoor air quality and crew health. Characteristics of studies included in this narrative review, structured according to a PECO(TS) framework. For each study, the table reports the study design (TS), population (P), exposure to submarine indoor air or related confined-environment conditions (E), exposure characterization (measured, estimated, or assumed), comparator group when available (C), health outcomes (O), main findings, and key limitations or sources of bias. This table is provided to ensure transparency of study selection and data extraction and to support the qualitative synthesis presented in the main text. Abbreviations: PECO(TS), Population–Exposure–Comparator–Outcomes (Study design); P, Population; E, Exposure; C, Comparator; O, Outcomes; TS, Study design; SMR, Standardized Mortality Ratio; CV, Cardiovascular; CO_2, carbon dioxide; VOCs, volatile organic compounds.

Reference	Country/context	Study design (TS)	Population (P)	Exposure (E)	Exposure characterization	Comparator (C)	Outcomes (O)	Outcome category	Main findings	Limitations / bias	Relevance
[2]	Military submarines	Narrative review	Submarine personnel	Confined submarine atmosphere	Assumed (theoretical)	None	Renal disorders; bone metabolism	Metabolic / renal	Hypothesized renal and phosphocalcic disturbances	Very old data; speculative; no epidemiology	Low
[33]	US Navy	Retrospective cohort	68,475 submariners	Submarine duty	Assumed	General population (SMR)	All-cause and CV mortality	Mortality / cardiovascular	No excess mortality compared with general population	Strong healthy worker effect; exposure not quantified	Moderate
[47]	Norwegian Navy	Registry-based cohort	2,663 submariners	Submarine service	Assumed	General population (SMR)	Cancer incidence; cause-specific mortality	Cancer / mortality	No excess cancer risk; reduced overall mortality	Exposure misclassification	Moderate
[7]	Military submarines	Observational	Active submariners	Chronic elevated CO_2	Measured (CO_2)	Internal comparison	Sleep-related respiratory disturbances	Respiratory / neuro	High CO_2 associated with sleep breathing disorders	Limited sample size	Moderate
[31]	US Navy	Observational	Submariners	Diesel exhaust during misstart events	Measured (incident-based)	None	Respiratory irritation	Respiratory	Documented acute diesel exhaust exposure	Incident-based; no long-term follow-up	Moderate
[16]	US Navy	Descriptive cohort	Submariners	Submarine service	Assumed	Surface naval personnel	Asthma prevalence	Respiratory	Higher asthma prevalence reported	Diagnosis based on records	Moderate

[17]	Italian Navy	Cross-sectional	Submariners	Submarine indoor air	Assumed	None	Respiratory symptoms	Respiratory	Indoor air associated respiratory complaints	Limited exposure data	Low–moderate
[38]	Netherlands Navy	Cross-sectional survey	742 veteran submariners	Submarine occupational environment	Assumed	General population	Self-reported health and vitality	General health	Higher vitality scores vs controls	Self-report; selection bias	Moderate
[34]	Republic of Korea Navy	Cross-sectional	590 naval personnel	Submarine service	Assumed	Surface naval personnel	Multimorbidity	General health	Higher multimorbidity in submariners	Exposure not quantified	Moderate
[11]	US Navy	Experimental	Submariners	Acute CO_2 exposure (0.06–1.5%)	Measured	Within-subject	Decision-making performance	Neurocognitive	No impairment observed	Short exposure duration	Moderate
[9]	Confined indoor environments	Experimental	Healthy adults	Elevated indoor CO_2 and co-varying VOCs	Measured	Lower CO2 condition	Decision-making performance	Neurocognitive	Reduced decision performance at higher CO_2/VOCs	Non-submarine setting	Moderate
[5]	Closed habitats	Narrative review	Submarine/space crews	Human-emitted VOC mixtures	Measured/assumed	None	Breathomics and exposure profiles	Biomarkers	Human metabolism major VOC source	Narrative synthesis	Moderate
[6]	Military submarines	Observational	Submarine crews	Confined humid environment	Measured (microbiology)	None	Airborne microbial flora	Bioaerosols	Presence of bacteria and fungi	Old data	Low
[22]	US Navy	Retrospective review	Submarine crew	Confined environment during COVID-19	Measured	None	SARS-CoV-2 transmission	Infectious	High transmission risk in confined air	Pandemic-specific context	Moderate
